# Supplementary material for: Development of a new largely scalable in vitro prion propagation method for the production of infectious recombinant prions for high resolution structural studies
Source: PLoS Pathog. 2019 Oct 23;15(10):e1008117. doi: 10.1371/journal.ppat.1008117 (PMC6827918; doi:10.1371/journal.ppat.1008117)
Supplement: S1 Fig — Photograph on the left shows the rack designed with 96 wells for 0.2 ml PCR tubes on the shaker used for PMSA reactions. The photograph on the right shows the rack with 6 horizontal cells for 5 ml tubes suitable for the same shaker. Both racks were designed ad hoc and 3D-printed (I+3D). Racks for any desired tube can be designed and printed making PMSA a highly versatile method for misfolded protein production. (PDF) [file ppat.1008117.s001.pdf]

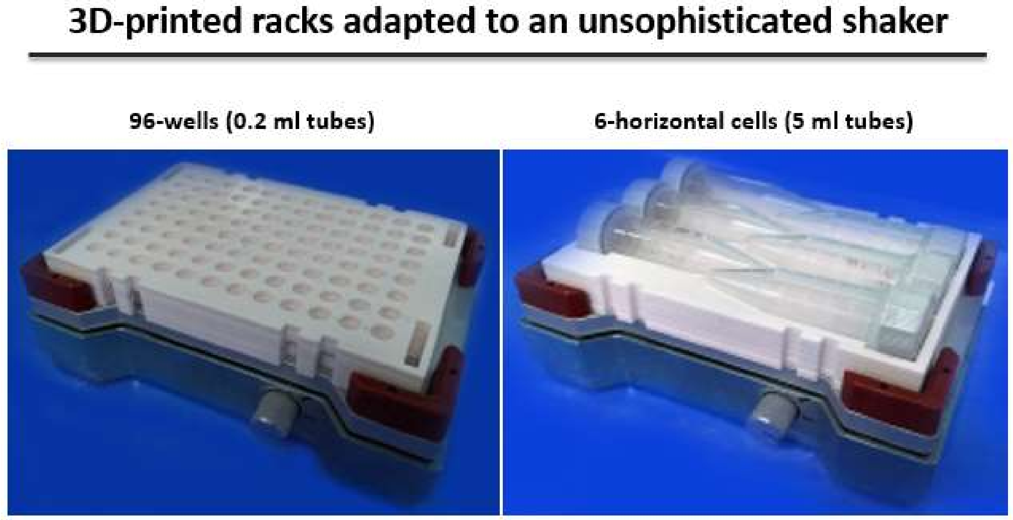

Fig. S1. Shaking system and *ad hoc* designed racks for different tube sizes. Photograph on the left shows the rack designed with 96 wells for 0.2 ml PCR tubes on the shaker used for PMSA reactions. The photograph on the right shows the rack with 6 horizontal cells for 5 ml tubes suitable for the same shaker. Both racks were designed *ad hoc* and 3D-printed (I+3D). Racks for any desired tube can be designed and printed making PMSA a highly versatile method for misfolded protein production.
