# Supplementary material for: Development of a new largely scalable in vitro prion propagation method for the production of infectious recombinant prions for high resolution structural studies
Source: PLoS Pathog. 2019 Oct 23;15(10):e1008117. doi: 10.1371/journal.ppat.1008117 (PMC6827918; doi:10.1371/journal.ppat.1008117)
Supplement: S2 Fig — 10% brain homogenates from TgVole (1x) (I109I) mouse and bank vole 109I were diluted 1:16, 1:32, 1:64 and 1:128 and analyzed by Western blot using monoclonal antibody D18 (1:5,000). The PrP expression levels of TgVole (1x) were equal to PrPC levels in bank vole brain based on signal intensity. No significant differences were observed in the electrophoretic migration patterns. Mw: Molecular weight. (PDF) [file ppat.1008117.s002.pdf]

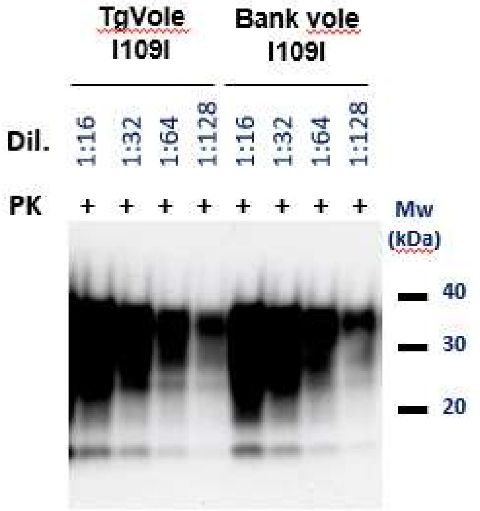

Fig. S2. PrP expression levels in TgVole (1x) animals compared to PrP expression levels of bank vole I109 by Western blot. 10% brain homogenates from TgVole (1x) (I109I) mouse and bank vole 109I were diluted 1:16, 1:32, 1:64 and 1:128 and analyzed by Western blot using monoclonal antibody D18 (1:5,000). The PrP expression levels of TgVole (1x) were equal to PrP^C levels in bank vole brain based on signal intensity. No significant differences were observed in the electrophoretic migration patterns. Mw: Molecular weight.
