# Supplementary material for: Development of a new largely scalable in vitro prion propagation method for the production of infectious recombinant prions for high resolution structural studies
Source: PLoS Pathog. 2019 Oct 23;15(10):e1008117. doi: 10.1371/journal.ppat.1008117 (PMC6827918; doi:10.1371/journal.ppat.1008117)
Supplement: S1 Table — (PDF) [file ppat.1008117.s004.pdf]

S1 Table. L-seeded-PMSA protease K-resistant fragments identified by ESI-TOF.

Experimental mass	Sequence	Theoretical mass
9435.13	N153-S231	9436.44
9321.03	M154-S231	9322.34
9451.43	N153-S231 (MetSO)[1]	(+16)
9337.81	M154-S231 (MetSO)[1]	(+16)
6105.20	N97-E152	6106.93
	Q98-N153	6106.93
6219.46	N97-N153	6221.03
6088.28	N97-E152 (N-terminal aspartic succinimide)[2]	(-17)
	Q98-N153 (N-terminal pyroglutamic acid)[2]	(-17)
5991.26	N97-E153	5995.83
5974.18	N97-N153 (N-terminal pyroaspartate)[1]	(-17)

[1] Oxidation of Met to methionine sulfoxide (MetSO).
[2] https://abrf.org/delta-mass
