# Supplementary material for: Development of a new largely scalable in vitro prion propagation method for the production of infectious recombinant prions for high resolution structural studies
Source: PLoS Pathog. 2019 Oct 23;15(10):e1008117. doi: 10.1371/journal.ppat.1008117 (PMC6827918; doi:10.1371/journal.ppat.1008117)
Supplement: S3 Fig — Proteinase K-digested and undigested L-seeded-PMSA were subjected to ultracentrifugation at ~200,000 g through a continuous sucrose gradient ranging from 10 to 80% (w/v) with 0.2% sarkosyl to determine the size distribution of total PrP and PrPres assemblies by Western blot. The equivalent of 2 μl of each fraction of the gradient were loaded for the undigested sample, while for PK-digested sample, fractions were precipitated with methanol loading the equivalent to 60 μl, thus PK-digested gel shows 30-fold higher sample amount than the gel of undigested fractions. Briefly, the sedimentation pattern shows total absence of monomeric PrP in the top fractions and a continuum in the aggregate size from fractions containing around 30% to 80% sucrose for the untreated sample and from around 40% to 80% sucrose for the PK-digested sample, suggesting predominance of large PK-resistant assemblies in the sample. This indicates the presence of a heterogeneous size assemblies but does not reveal clearly different majoritarian populations that could point towards the existence of distinct assembly types regarding tertiary structure. Developed with Saf83 mAb (1:400). PK: Proteinase K, MW; molecular weight marker. (PDF) [file ppat.1008117.s003.pdf]

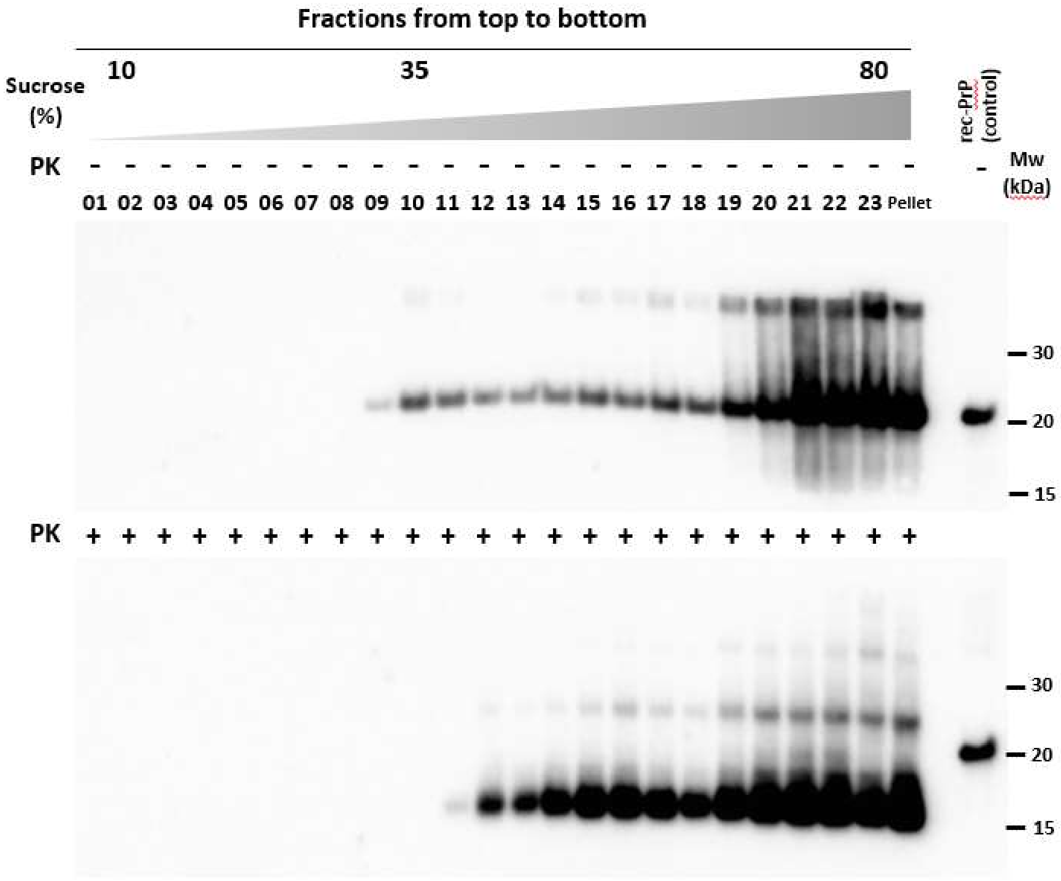

Fig. S3. Sedimentation velocity analysis of L-seeded-PMSA by ultracentrifugation through a continuous sucrose gradient. Proteinase K-digested and undigested L-seeded-PMSA were subjected to ultracentrifugation at ~200,000 *g* through a continuous sucrose gradient ranging from 10 to 80 % (w/v) with 0.2 % sarkosyl to determine the size distribution of total PrP and PrP^{res} assemblies by Western blot. The equivalent of 2 µl of each fraction of the gradient were loaded for the undigested sample, while for PK-digested sample, fractions were precipitated with methanol loading the equivalent to 60 µl, thus PK-digested gel shows 30-fold higher sample amount than the gel of undigested fractions. Briefly, the sedimentation pattern shows total absence of monomeric PrP in the top fractions and a continuum in the aggregate size from fractions containing around 30 % to 80 % sucrose for the untreated sample and from around 40 % to 80 % sucrose for the PK-digested sample, suggesting predominance of large PK-resistant assemblies in the sample. This indicates the presence of a heterogeneous size assemblies but does not reveal clearly different majoritarian populations that could point towards the existence of distinct assembly types regarding tertiary structure. Developed with Saf83 mAb (1:400). PK: Proteinase K, MW; molecular weight marker.
